# “*I just assumed that was the same as colon cancer.*” Lack of knowledge about anal HPV infection, anal cancer, and anal cancer screening in older MSM: a qualitative study

**DOI:** 10.1186/s12889-025-25202-w

**Published:** 2025-11-21

**Authors:** Catherine Nguyen, Danielle Miguel, Christopher Scott Weatherly, Sahai Burrowes, Jessica Lopez Jimenez, Ryan Gonzalez, Joel M. Palefsky, Alexandra L. Hernandez

**Affiliations:** 1 College of Osteopathic Medicine, Touro University California, Vallejo, CA USA; 2 Public Health Program, College of Education and Health Sciences, Touro University, Vallejo, CA USA; 3 University Medical Center of Southern Nevada, Las Vegas, NV USA; 4 Department of Medicine, Division of Infectious Diseases, University of California, San Francisco (UCSF), Box 0654, 513 Parnassus Ave, Room S420, San Francisco, CA 94143 USA

**Keywords:** MSM, HIV, Qualitative, Focus groups, HPV, Anal cancer, Health education, Knowledge

## Abstract

**Background:**

Antiretroviral treatment has allowed people living with HIV (PLWH) to live longer lives. A high proportion of PLWH are ≥ 50 years of age and identify as men who have sex with men (MSM); MSM who are living with HIV (MSMLWH) are at increased risk for anal cancer and anal human papillomavirus (HPV) infection, its causal agent. We explored knowledge of anal cancer, HPV infection and anal cancer screening using qualitative methods.

**Methods:**

Five focus group discussions were conducted with 21 MSMLWH and 11 MSM not living with HIV, ages 50–75 years, in San Francisco, California. Data were analyzed using a thematic analysis approach employing a mix of inductive and deductive codes.

**Results:**

We identified three main themes and eight sub-themes that characterized participants’ understanding of HPV and anal cancer: (1) *Lack of knowledge*,* including lack of knowledge of HPV/anal cancer; lack of knowledge of HPV vaccine; lack of knowledge that colonoscopy does not screen for anal cancer; and a perceived lack of knowledge about anal cancer in health care providers;* (2) *Personal experience with HPV and anal cancer*; and (3) *Awareness of HPV and anal cancer*,* including general awareness and awareness through celebrities or the media.* There were important knowledge gaps among MSM aged 50 and older about HPV infection and anal cancer, including a misunderstanding that colorectal cancer screening can detect anal cancer. Increasing health provider knowledge and engaging the media or celebrities in health education campaigns could be effective strategies to increase awareness.

**Conclusions:**

The recently released federal screening guidelines for anal cancer recommend screening all MSM and transgender women over the age of 35 who are living with HIV. Public health professionals should be aware of knowledge gaps among older MSM that pose barriers to screening. Given that our participants are in the age group where colorectal cancer screening is routinely recommended, our findings suggest that awareness of anal cancer risk may not be sufficient to motivate screening if patients believe they have already been screened. Education campaigns should also teach the difference between anal cancer screening and colorectal cancer screening.

## Background

Although anal cancer is rare in the general population (0.8 per 100,000 people), cases have been rising significantly over the last two decades [1]. Certain groups have a much higher risk of acquiring human papillomavirus (HPV), the virus that causes most anal cancers, developing HPV-associated high-grade squamous intraepithelial neoplasia (HSIL)—the anal cancer pre-curser, and developing anal cancer [1]. The incidence of anal cancer among men who have sex with men (MSM) is 19 per 100,000 people, and the population with the highest incidence of anal cancer is MSM who are living with HIV (MSMLWH) at 85 per 100,000 people [2]. Anal HPV infection and HSIL prevalence are high among MSMLWH (80–90% and 34%, respectively) compared to MSM who do not have HIV [3, 4]. While effective antiretroviral therapy (ART) has reduced the occurrence of many AIDS-defining illnesses, this transformative medical intervention has not impacted anal cancer, and the incidence among people living with HIV (PLWH) remains high [5]. As with many cancers, the incidence of anal cancer increases with age both for those living with those without HIV [6]. 

As almost half of PLWH are now over 50 older PLWH may be a particularly vulnerable population for anal cancer [7]. The public health community needs to understand the knowledge, attitudes, beliefs, and needs of older communities of PLWH and MSMLWH to design appropriate prevention initiatives. Initiatives could include secondary prevention efforts such as screening and removal of HSIL lesions, as demonstrated by the recent Anal Cancer HSIL Outcomes Research (ANCHOR) randomized controlled trial [8]. 

Previous research shows that while these preventative initiatives are acceptable to PLWH, lack of awareness of HPV can prevent individuals from seeking these services [9]. Studies have also found that more familiarity with HPV infection was associated with increasing self-perceived risk in MSM [9]. Although increased knowledge or understanding does not directly ensure improved health behaviors and outcomes, it promotes self-efficacy, risk perception and values reflection, which are key to effective health education interventions as part of the health belief model [10]. Thus, assessing older MSMs’ understanding of HPV and anal cancer is a necessary part of reducing the burden of anal HPV infection-related morbidities and mortalities for this at-risk population.

The literature on the gaps in HPV and anal cancer knowledge within the general population demonstrates that anal cancer knowledge and risk perception is low overall. HPV awareness is commonly framed in a women’s health and cervical cancer context, and this gendered association of HPV is also pervasive among MSMLWH [11–16]. This paper presents results on participant’s anal cancer knowledge and awareness collected as part of a qualitative study using focus group discussions [17]. We sought to characterize the knowledge of anal cancer, anal HPV infection, anal cancer screening methods, among MSM ages 50 and over.

## Methods

The methods for this study have been previously published [17]. The qualitative study was designed to obtain feedback on study recruitment methods for the aging MSM population. The study also involved introductory questions assessing the participants’ existing knowledge of HPV infection, anal cancer, HPV vaccination and screening recommendations. Five focus group discussions (FGDs) were facilitated between June and November 2018. A paper survey was administered before beginning the FGD, in which individuals answered open-ended demographic questions asking them to self-report age, gender identity, sexual orientation, HIV status, and race/ethnicity. All study procedures were approved by the University of California, San Francisco (UCSF) Institutional Review Board. Written informed consent was obtained from all participants before data collection. The COnsolidated criteria for REporting Qualitative research (COREQ) checklist was used to report the research findings [18]. 

### Participant recruitment

Participants were recruited from the UCSF Anal Neoplasia Research and Education Center (ANCRE). Individuals were eligible for the original study if they reported being over 50 years old and identified as men or transgender individuals who have sex with men. The pool of participants was purposively sampled to balance HIV status and age group. We initially reached out via email to potential participants known to study staff. Thirty-seven individuals were scheduled for a FGD through a follow-up phone call. Five of these individuals did not present for their scheduled FGD, and we did not collect information on gender identity during recruitment or why they were unable to attend. Our remaining total of thirty-two participants were enrolled and completed the entirety of the FGD.

### Focus group procedures

Facilitators oriented participants by reading a script containing introductory information on HPV and anal cancer, as well as general guidelines for a respectful discussion. A guide of nine open-ended discussion points was used to direct the conversation (the full focus group guide has been previously published) [17]. Three questions directly assessed anal cancer awareness and were asked at each FDG. These questions were: “Have you ever heard of anal cancer? What comes to mind when you think of anal cancer? How do you think men and trans-people in our target age group would react to the anal exams that we described?”

Five FGDs were conducted in a private conference room in the same building as the ANCRE clinic. Each enrolled individual was scheduled for and participated in only one FDG, which lasted 90 to 120 min. Only the scheduled participants and designated researchers were present at the FGDs. Data were collected via audio recording and handwritten notes by facilitators. FGD participants were not provided transcripts or findings. Participants were reimbursed for their time and travel for study with a meal (sandwich or salad, chips and drink) and a cash payment of $25.

### Research team and reflexivity

A table of researcher characteristics was published previously [17]. At all FGDs, RG (senior research coordinator, non-binary) was the primary focus group facilitator, and ALH (principal investigator, cis-female) supported facilitation. Before focus group discussions, researchers introduced themselves and described their study roles to participants. RG knew many of the focus group participants from prior ANCRE research studies he coordinated. ALH had previously worked with one participant in FGD 2. CSW, JLJ, SB, CN, and DM did not attend FGDs and only reviewed de-identified data.

### Data analysis

We used thematic analysis supported by Dedoose software to apply codes and analyze data [19, 20]. We used a mixed, inductive, and deductive coding strategy using both a priori codes drawn from our FGD guide and new codes that emerged from the data during analysis. All themes were developed after focus groups were carried out via analysis during coding.

All FGD audio recordings were professionally transcribed and read through manually for coding. ALH and CSW independently coded FGD 1, compared coding results, and confirmed consistent coding methods. After ensuring consistency, CSW and JLJ independently coded and reviewed the five FGDs’ results. During these reviews, new codes and themes were discovered and updated. ALH resolved any disagreements in coding. CN and DM additionally coded all five transcripts for knowledge-related themes, including knowledge about HPV infection, HPV vaccination, anal cancer and anal cancer screening. ALH verified new knowledge codes. FGDs 4 and 5 produced no new themes, and researchers determined that thematic analysis had reached saturation [21, 22]. 

## Results

### Participant characteristics

Demographics of our study participants are summarized in Table 1 by individual focus group groups and as total study population. Thirty-two individuals participated in the five focus group discussions. All participants self-identified their gender identity as cisgender and their sexual orientation as MSM or bisexual. Over half of our participants were living with HIV (65%). About 34% percent were 50–59 years old (*n* = 11), 43% were 60–69 years old (*n* = 14), and 22% were 70 + years old (*n* = 7). Race and ethnicity data were collected in an open-ended field. Most participants, 75% (*n* = 24), described their race/ethnicity as “White.” 16% (*n* = 5) self-reported as “Latino,” “Mexican/White,” or “Hispanic/White.” 6% (*n* = 2) self-reported as “Black” or “Black Mix.” 3% (*n* = 1) self-reported as “Asian/White.” All participants who reported a non-White or mixed White/non-White race/ethnicity were living with HIV.

**Table 1:** Participant characteristics by focus group discussion and HIV status (MSMLWH *n* = 21, HIV-negative MSM *n* = 11)

	FGD 1 (*n* = 5)		FGD 2 (*n* = 6)		FGD 3 (*n* = 7)		FGD 4 (*n* = 7)		FGD 5 (*n* = 7)		Total FGDs (*n* = 32)	
	MSM LWH	HIV(-) MSM	MSM LWH	HIV(-) MSM	MSM LWH	HIV(-) MSM	MSM LWH	HIV(-) MSM	MSM LWH	HIV(-) MSM	MSM LWH	HIV(-) MSM
Age												
50–59	1	3	1	1	2	1	1	0	0	1	5	6
60–69	0	0	1	3	3	0	2	1	3	1	9	5
70+	1	0	0	0	1	0	3	0	2	0	7	0
Race/ethnicity												
White	2	2	0	4	4	1	4	1	4	2	14	10
Latino/Hispanic/Mexican	0	0	1	0	1	0	2	0	1	0	5	0
Black or Black Mixed	0	0	1	0	1	0	0	0	0	0	2	0
Asian or Asian Mixed	0	1	0	0	0	0	0	0	0	0	0	1
Declined to Respond	0	0	0	0	0	0	0	0	0	0	0	0

Some participants identified with more than one race/ethnicity. All participants identified as cis-gendered

*FGD* Focus Group Discussion

*MSM* Men Who Have Sex with Men

*LWH* Living with HIV

### Thematic analysis

Three major themes related to anal cancer knowledge were identified from the five FGDs: “Lack of Knowledge of HPV Infection, HPV Vaccination, and Anal Cancer,” “Personal Experience with HPV/Anal Cancer,” and “Awareness of HPV/Anal Cancer.” Table 2 presents each theme into eight sub-themes with a description, example quote, and theme frequency in each focus group.

**Table 2:** HPV and anal cancer knowledge identified from thematic analysis of focus group discussions

Theme		Description	Example Quote	Theme frequency by Focus Group Discussion (FGD)					
Sub-Theme				#1	#2	#3	#4	#5	Total
1. Lack of knowledge of hpv infection, HPV vaccination, and anal cancer									
1.1 Lack of Knowledge of HPV Infection or Anal Cancer		Made incorrect statements about anal cancer, anal HPV, or anal cancer screening, or stated that they did not know when asked questions to assess knowledge.	“I feel like [I] have a lot of confusion, have interest about anal cancer and HPV, but I feel like I haven’t really had clear sources of what tests can be done and what information can be gotten from those tests.” *–FGD 1*	10	8	6	11	4	39
1.2 Lack of Knowledge of HPV Vaccine		Made incorrect statements about HPV vaccination or stated that they did not know when asked questions to assess knowledge	“What I know about HPV is there is a vaccine for it, correct? So would a person be better off to go get a vaccine than to join your study?” *–FGD 4*	0	3	0	2	0	5
1.3 Lack of Understanding of Colon Cancer/Colonoscopy vs. Anal Cancer		Expressing a lack of understanding about the difference between colonoscopy and anal cancer screening or colon and anal cancer.	“I guess to be honest I am not really clear either on like… if you’re having a colonoscopy, will it show there?” *–FGD 1*	2	2	5	2	1	12
1.4 Perceived Lack of Knowledge about Anal Cancer and Anal Cancer Screening in Healthcare Providers		Experiencing encounters with providers demonstrating their lack of knowledge of anal cancer/anal cancer screening	“So, in talking about the lack of knowledge about anal cancer, it strikes me even among doctors, right. It strikes me that there needs to be small booklet or pamphlet called, ‘How to take care of yourself as a gay man after 50’.” *–FGD 4*	1	5	0	1	0	7
2. Personal experience with HPV/anal cancer									
2.1 Personal Experience with HPV Infection		Stating correct knowledge through the individual or someone close to them has or had HPV, warts, an anal pap, or an HPV vaccine.	“And I had HPV as a teenager and it reoccurred in my 30s, and I used to go in for an annual screening, … but some years back they talked about anal paps, pap smears and I was really curious about what that—what kind of information, so I feel like I don’t have knowledge about detection and prevention” *–FGD 1*	4	10	2	1	4	21
2.2 Personal Experience with Anal Cancer		Stating correct knowledge that the individual or someone close to them has or had anal cancer.	“I have a good friend who had a horrible death with anal cancer and that was really maybe a year or so before I had learned about your study. And so that was really good to know, but to have witnessed that process was horrifying and very scary, and so I hope to have more people aware of having the testing done so they don’t go through something like that, because hopefully, you have survivable cancer.” *–FGD 3*	0	2	3	0	1	6
3. Awareness of HPV/Anal cancer									
3.1 Awareness of HPV/Anal Cancer		Demonstrating awareness about HPV, anal cancer, dysplasia, HPV vaccines, or warts.	“[Tops] just as with heterosexual woman; you don’t have to have engaged in anal sex, because of vaginal or oral sex, they have gotten anal cancer.” *–FGD 1*	6	8	5	1	1	21
3.2 Awareness of HPV/Anal Cancer through Celebrities or the Media		Expressing awareness of HPV or HPV-associated cancers through celebrities or the media.	“Yeah, and I think, you know, also to your point, I think until Farrah Fawcett, I just assumed that was the same as like colon cancer, there wasn’t much difference other than maybe location, whether it’s just sort of a name for a different thing and I think they are different” *–FGD 3*	5	4	5	1	3	18

### Theme 1: lack of knowledge of HPV Infection, HPV vaccination, and anal cancer

#### Subtheme 1.1: lack of knowledge of HPV infection or anal cancer

A strong theme that emerged was that most FGD participants lacked knowledge about anal HPV infection, anal cancer and screening for anal cancer. Many participants expressed that they had never heard about anal cancer until they encountered our clinic. There was agreement among most participants that they did not know who was at risk for anal cancer or which factors could increase their risk for anal cancer. Some men did not know that the risk of anal cancer was higher in MSM and among PLWH compared with the general population.

*“So it is higher in a gay man or is there*,* is a statistic[al variation between] gay men and straight men*,* …?” –FGD 2*.

*“[Anal cancer] like there used to be an assumption that only bottoms get HIV*,* we have those biases and prejudice*,* they are not rational*,* and they are not true*,* but I think there is still some bottom shaming in our culture.” –FGD 1*.

Participants also did not know how one would get screened for anal cancer.

*“So I feel like I have a lot of confusion*,* [I] have interest about anal cancer and HPV*,* but I feel like I haven’t really had clear sources of what tests can be done and what information can be gotten from those tests.” –FGD 1*.

There was also a lack of knowledge about the symptomatic presentation of anal HPV infection and anal cancer. Many participants were unsure if anal warts, polyps, lesions, or rectal bleeding were associated with HPV infection or anal cancer.

*“A lesion and a wart and polyp*,* are they different things?” –FGD 1*.

Some men also expressed being unaware that HPV could present without symptoms. They felt that if they knew that anal HPV could present asymptomatically, it would motivate them to pursue early preventative screening. There was also some uncertainty about how long an individual would test positive for HPV.

*“…there is no concept so far as I know in a gay community or amongst any people at all that you can have any kind [of] disease asymptomatically.” –FGD 2*.

There was also a lack of knowledge of other types of cancers that can develop from HPV infection, particularly penile cancer.

*“I think I was confused*,* like*,* is that penile cancer*,* which is very rare? Was it that—does it cause both?” –FGD 3*.

#### Subtheme 1.2: lack of knowledge of HPV vaccine

Participants also expressed uncertainty regarding the role of HPV vaccination in the prevention of anal cancer. Most participants were not aware of the causal association between HPV and anal cancer. While many individuals had heard of the HPV vaccine through the media and word of mouth, there was skepticism about its usefulness in men.

*“Actually*,* I didn’t even think it was for men. I think it was for girls when it first came out cause I was like*,* this is new. I had never heard of [the HPV vaccine].” –FGD 2*.

They also were confused about receiving the HPV vaccine and whether obtaining it obviated the need to screen for anal cancer.

*“What I know about HPV is there is a vaccine for it*,* correct? So would a person be better off to go get a vaccine than to join your study?” –FGD 4*.

Some believed that the HPV vaccine was recommended as a preventative measure, even at ages over 50 years.

#### Subtheme 1.3: lack of understanding of colon cancer/colonoscopy vs. anal cancer

There was also much confusion regarding the differences between screening for anal cancer and having a colonoscopy–which screens for colorectal cancer. Participants believed that colon cancer screening was important, and many participants had had a colonoscopy or a stool-based colon cancer screening test. However, many believed incorrectly that a colonoscopy also screens for anal cancer.

“*… I just assumed that was the same as like colon cancer*,* there wasn’t much difference other than maybe location*,* whether it’s just sort of a name for a different thing. And I think they are different*,* again I consider myself reasonably educated on these kind of things*,* but if you ask me to really draw a distinction*,* it will be hard to say and so there is a way in which I figure well*,* when I am 50*,* I am going to get the colonoscopy or the screening and that will catch any problems…” –FGD 3*.

Participants also did not know if newer colon cancer screening tests that act as alternatives to colonoscopy and would screen for anal cancer.

*“…instead of having a colonoscopy*,* this last month I did something called Cologuard which is sort of a new test where you collect a stool specimen and they send it in and then folks have seen that ads on TV*,* and I wondered would that pick up on the anal cancer or is that specific to colon cancer?” –FGD 3*.

Participants were also unsure about the timeline for anal cancer screening and if it aligned with colon cancer screening. They did not know when preventative screenings should be initiated with their provider and how often after the initial visit they should continue checking.

Focus group participants also discussed conflating the colon cancer screening experience with the anal cancer screening experience because they both involve the gastrointestinal tract. They falsely believed that high-resolution anoscopy (HRA) was an equally involved process that required a full-day commitment.

*“Yeah. It may be that people because they don’t know of colonoscopies which is a big deal and it does take whole day and there is prep and you’re not doubt in all that stuff. They may equate this and figure anything that’s going through the back doors going to have the same issues right. In which case they say I’m not doing that but if it’s clear that it’s just a 20-minute [exam].” –FGD 4*.

#### Subtheme 1.4: Perceived lack of knowledge about anal cancer and anal cancer screening in healthcare providers

Participants also felt that their healthcare providers did not have sufficient knowledge about their risk for anal cancer, the availability of anal cancer screening, or how to refer them for screening.

*“It took me a long time*,* I did some research online and it seemed that I should have anoscopy and Pap smear*,* but I went through three to four doctors first who did not know what it is talking about. One of them offered to do it*,* but I thought she said what we could do kind of record*,* that finally a doctor and I went him and said I had genital warts and he said*,* oh what will do Pap smear and anoscopy. I knew that I was at the right place.” –FGD 2*.

There was also a feeling among participants that healthcare providers who were straight (heterosexual) may not understand the needs of gay men.

*“I have to say I went… I was going to all these doctors who were straight and it didn’t… that didn’t bother me. What bothered me was that I kept getting the same puzzled look*,* and the [Unclear] was I thought I have to go somewhere else when this was happening.” –FGD 2*.

### Theme 2: personal experience with HPV/anal cancer

#### Subtheme 2.1: personal experience with HPV infection

Some participants did have correct knowledge about HPV or its consequences through direct experience that they, or a friend or family member they knew, had personally had HPV infection and cancers caused by HPV, like cervical cancer.

*“You know my mother had cervical cancer and it was caused by HPV when she was… and I remember I was doing research about it*,* and I remember one of the first things that came up about what caused it was like having lots of sex as a teenager. ” –FGD 2*.

Other participants had had HPV infections themselves in the past or had general knowledge about HPV infection being common.

*I have known since the 80s that we have all had HPV and not the—the stories aren’t very happy stories*,* you know*,* their treatments are pretty traumatic and frustrating and confusing—what is the outcome and whether you are really—are you ever cleared of HPV*,* you know when to relax.” –FGD 1*.

#### Subtheme 2.2: personal experience with anal cancer

Other participants had correct knowledge about anal cancer through experiences. Several participants shared their own experiences with anal cancer or anal cancer screening or the experiences of close family or friends with anal cancer. There were a range of reported experiences, from complete recovery to death.

*“I have been checked for anal cancer twice and have another appointment in January at Kaiser.” –FGD 5*.

*“I have a good friend who had a horrible death with anal cancer*,* and that was really*,* maybe*,* a year or so before I have learned about your study. And*,* so*,* that it was really good to know but to have witnessed that process was horrifying and very scary*,* and so I hope to have more people aware of having the testing done so they don’t go through something like that because hopefully you have a survivable cancer.” –FGD 3*.

*“I had a friend [name of friend] that had and got over anal cancer as well*,* so saw it firsthand*,* but pulled through*,* is fine now so. Yeah*,* she went through a whole course to deal with it*,* and she just happened to start bleeding suddenly*,* and that’s how she was brought aware of the whole issue*,* so it was totally sudden for her but she pulled through.” –FGD 3*.

### Theme 3: awareness of HPV/anal cancer

#### Subtheme 3.1: awareness of HPV/anal cancer

While most of the discussions centered around a lack of knowledge, participants did report some general awareness regarding HPV and anal cancer. Some were aware of the association between HPV and anal cancer.

*“Easy to miss early detection for sure*,* you know*,* I have also heard it could be associated with HPV and that just like with cervical cancer*,* an anal HPV can increase risks for anal cancer.” –FGD 1*.

A few participants knew that men identifying as gay may have an increased risk for anal cancer.

*“If you put it out there more saying not just gay men. You know we get it because that is what we know about this. That it is a little too polite being talked about. Be blunt about it you know. You know this is not just about gay men. All men should be checked*,* put the emphasis on that - all men*,* not men who have sex with other [men].” FGD 2*.

Given that each of these participants had previously interacted with the ANCRE clinic, it is unsurprising that many expressed awareness of the availability of anal cancer screening and treatment.

*“I’ve heard about it maybe should be regularly at the age of 55*,* you should have regular yearly screenings that’s what I meant.” –FGD 4*.

Many participants who had correct knowledge also discussed their direct experience with the ANCRE Clinic when discussing correct knowledge about anal cancer, anal cancer screening, or HPV.

#### Subtheme 3.2: awareness of HPV/anal cancer through celebrities or the media

Several participants had heard about HPV-associated cancers through reports in the media of celebrities having had anal cancer.

“Farrah Fawcett … She was the only famous person that I am aware of died of it and talked about it while she was dying of it and kind of went public with it.” –FGD 3.

“… I think everybody is aware of that when it comes to women and the link to cancers being advertised and you have Michael Douglas with the throat cancer” –FGD 3.

Participants also mentioned the “Desperate Housewives” actress. While they did not know her by name (Marcia Cross), they did know that she had been diagnosed with anal cancer.

## Discussion

### HPV and anal cancer knowledge

We found a surprising lack of knowledge and awareness about anal cancer in our FGDs. All men in our study had had contact with the UCSF ANCRE Clinic, either as a clinic patient or had screened for or participated in a research study at the ANCRE Clinic. Most men with anal cancer awareness reported that they learned about it through contact with the UCSF ANCRE clinic and associated anal cancer studies. Even among those men, there was an important lack of knowledge about what causes anal cancer and who is at risk. Given that this specialized population isn’t representative of most older MSM, the lack of knowledge that we found is likely a conservative finding. It is to be expected that MSM who are not in contact with anal cancer clinics have even less understanding of their anal cancer risk and the benefits of screening than our study participants. Other studies have found a similar lack of knowledge about the connection between HPV and anal cancer; they have also found a lack of knowledge of risk factors for HPV infection. Grace et al. found that MSMLWH in their study had no prior knowledge of the health effects of HPV infection [16]. A 2018 review also established that MSMLWH had limited knowledge of the connection between HIV, HPV, and anal cancer [23]. 

Understandably, many of our participants, all over the age of 50 years, believed that colonoscopy or other screening methods for colon cancer also screened for anal cancer. While there is a physiological difference between the anus and the rest of the colon, lay individuals will likely not know that there is a difference or that cancers at these two locations can arise from different risk factors and require different screening methods for detection [24, 25]. Even if participants had heard of anal cancer, they believed that their colon cancer screening was also screening for anal cancer. Increasing education about anal cancer risks may be insufficient if people understand that they are at risk but believe they have been screened during their colon cancer screening. This misconception is particularly significant for MSM over age 50, who are in the age group where regular screening for colon cancer is recommended [25]. However, this poor understanding provides an opportunity to increase awareness and utilization by pairing anal cancer screening with colorectal cancer screening.

According to the National Health Interview Survey (NHIS), 59% of individuals aged 45 years and older were up to date on colorectal cancer screenings (CRC) in 2021 [26]. For those aged 50 and older, interventions are in place to screen those present in large-scale institutions. For example, Kaiser Permanente Northern California identifies patients over 50 and reminds them to be screened for colon cancer [27]. Aside from the reminder, Kaiser offers an online screening tool that educates patients about the screening, what options exist, the patient’s feelings towards the screening, potential decision towards screening, and a quiz to assess if the knowledge was understood correctly [27]. This screening serves as an essential intervention that normalizes and educates patients on colorectal cancer. Since this intervention and others like it are commonplace, patient education can be expanded to include anal cancer, for example, by including pamphlets sent with at-home screening kits. Pamphlets can explain the differences between anal and colorectal cancer and encourages patients to see their medical providers for a separate exam if they have any of the pertinent risk factors for anal cancer. Although women were not included in this study, this information could be beneficial to women as well as MSM, given more women than MSM are diagnosed with anal cancer each year [6]. Language and images used in these pamphlets or other education materials should be created in partnership with the communities they are meant to benefit, to ensure they are acceptable and sensitive to potential stigma from sexual behaviors, sexual orientation, or gender identity. A study of women’s knowledge of anal cancer risks and differences between anal cancer and colon cancer screening would also be beneficial in case their knowledge differs from that of men.

We also found a lack of knowledge about anal cancer screening and a perception that anal cancer screening is not being adequately addressed by healthcare providers, consistent with other studies’ findings [16, 23]. Because older MSM are a small group relative to the general population, large-scale community education campaigns–such as campaigns to promote mammograms for older women or to promote colonoscopies for all older individuals–may not be cost-effective [28]. However, targeted health education to providers may increase knowledge both among providers and their patients. Health education interventions targeting gerontologists, geriatricians, and healthcare providers who see PLWH may be helpful in this effort. Adding curricula at medical schools and physician assistant or nurse practitioner programs, as well as creating continuing medical education (CME) credits for practicing providers, could enhance healthcare practitioners’ knowledge about the risks of anal cancer and help them to differentiate colorectal cancer screening from anal cancer screening for their patients. There are existing CME courses on anal cancer and colorectal cancer, but none that integrate these two topics or among those aging with HIV [29, 30]. Improving provider education could include creating modules on “aging with HIV” or “Screening older MSM for anal cancer” for CME credits or clinical training programs.

Many patients who did have correct knowledge or awareness of anal cancer or HPV-associated anal disease did so because they or a friend or family member had had an experience with these illnesses or because they received information from the ANCRE Clinic. Some patients also had some correct knowledge about anal cancer or other HPV associated cancers through celebrities. Celebrities who speak out about their own stories with anal cancer (and other HPV-associated diseases) may help normalize anal disease in our culture and increase awareness. We have previously seen celebrity advocacy impact disease screening and awareness. As an example, Magic Johnson’s disclosure of his HIV status helped destigmatize HIV and increase awareness that anyone could test positive. His disclosure led to increased HIV testing overall and particularly in groups that previously did not believe themselves to be at risk for HIV, including women [31]. After the death of her husband from colon cancer, Katie Couric had a colonoscopy on live television to raise awareness and reduce stigma for colon cancer. The 19% bump in colonoscopies performed in the US was called “The Couric Effect” [32, 33]. Health education campaigns using either national or community celebrities discussing or recommending anal cancer screening may be another avenue to increase knowledge and awareness of all HPV-associated illnesses. Further studies could evaluate the usefulness of these health education campaigns.

### Limitations

Those who voluntarily took part in our FGDs might have had a higher level of knowledge regarding anal cancer and screening compared to average individuals in our target demographic because we recruited participants from the ANCRE clinic in San Francisco. This underscores the significance of our findings concerning the lack of anal cancer knowledge among aging MSM, as men who are not in contact with our clinic, particularly older men, are likely to be even less aware than our study participants. Another limitation of our study is the absence of female participants, who are also affected by anal HPV and anal cancer, given primary rationale for conducting this research was to facilitate recruitment for the parent study. Knowledge of this topic among aging women should also be assessed. Additionally, the demographics of our focus groups were unbalanced for certain factors. Our study would have benefited from more racially and ethnically diverse participants as a majority of our participants identified as “White,” and all participants who were people of color were PLWH. In some FGDs, MSMLWH outnumbered HIV-negative men by 6:1 or 5:1, so MSMLWH may have dominated the conversation. Additionally, our coding did not account for general or correct knowledge acquired during the FGD, and it is possible that FGD participants were informed by other participants or facilitators during discussions, evoking comments that were then coded as correct knowledge. The underlying knowledge may be less than what was found in our analysis.

## Conclusions

The US federal government recently released guidelines recommending that all MSMLWH and transgender women living with HIV over the age of 35 years be screened for anal cancer through high-resolution anoscopy [34]. The public health community should be aware of gaps in knowledge about anal HPV infection, anal cancer, and screening for anal cancer among aging MSM, particularly the incorrect belief that colorectal cancer screening will also detect anal cancer. A promising opportunity exists to partner with colorectal cancer screening programs to increase knowledge of risk factors for anal cancer and anal cancer screening programs. Additionally, interventions that educate providers who care for older MSM or PLWH may help distinguish colorectal cancer screening and anal cancer screening for patients. Finally, engaging celebrities in awareness campaigns for anal cancer screening may prompt older MSM to seek anal cancer screening.

## Data Availability

Data will be made available upon request. Please contact Alexandra Hernandez (alexandra.hernandez@ucsf.edu) to request access.

## Abbreviations

ANCHOR: Anal Cancer HSIL Outcomes Research
ANCRE: Anal Neoplasia Clinic, Research and Education Center
ART: Antiretroviral Therapy
CME: Continuing Medical Education
COREQ: COnsolidated criteria for REporting Qualitative research
CRC: Colorectal Cancer
FGD/FGDs: Focus Group Discussion(s)
HIV/AIDS: Human Immunodeficiency Virus/Acquired Immunodeficiency Syndrome
HPV: Human Papillomavirus
HRA: High Resolution Anoscopy
HSIL: High-grade Squamous Intraepithelial Lesion
MSM: Men who have Sex with Men
MSMLWH: Men who have Sex with Men Living With HIV
NHIS: National Health Interview Survey
PLWH: People Living With HIV
UCSF: University of California, San Francisco

## Code availability

The focus group code book and coding document are available from the corresponding author upon request.
